# Magnetic resonance imaging evaluation of the effectiveness of FemiCushion in pelvic organ prolapse

**DOI:** 10.1111/jog.15210

**Published:** 2022-02-28

**Authors:** Yukiko Nomura, Yoshiyuki Okada, Chie Nakagawa, Ippei Kurokawa, Miwa Shigeta, Hidefumi Fujisawa, Yasukuni Yoshimura

**Affiliations:** ^1^ Department of Female Pelvic Health Center Showa University Northern Yokohama Hospital Kanagawa Japan; ^2^ Department of Obstetrics and Gynecology Showa University Northern Yokohama Hospital Kanagawa Japan; ^3^ Department of Urology Showa University Northern Yokohama Hospital Kanagawa Japan; ^4^ Department of Radiology Showa University Northern Yokohama Hospital Kanagawa Japan

**Keywords:** conservative treatment, magnetic resonance imaging, pelvic organ prolapse, pessaries, quality of life

## Abstract

**Aims:**

FemiCushion (FC) is a supportive device for pelvic organ prolapse (POP), but its effectiveness has not been evaluated with imaging studies. This study utilized magnetic resonance imaging (MRI) to evaluate the anatomic changes induced by FC use in patients with severe POP.

**Methods:**

This prospective study examined patients with stage 3 or 4 POP who underwent treatment with FC and received a diagnostic MRI. Measurements were made in the midsagittal plane at rest and during straining with and without FC. The vertical distances from the lowest points of the anterior and posterior vaginal wall (A; P), uterine cervix or vaginal stump (C), and perineal body (PB) to the Pelvic Inclination Correction System line were measured, along with the lengths of the urogenital (UGH) and levator hiatus (LH).

**Results:**

Twelve patients were included in the study. The median age was 72 (range, 56–84) years. All reference points were positioned significantly higher with the FC than without the FC (median ΔA: 11 mm, *p* = 0.005; ΔC: 14 mm, *p* = 0.011; ΔP: 6 mm, *p* = 0.008; ΔPB: 7 mm, *p* = 0.002). Median UGH and LH lengths during straining were significantly shorter with the FC than without the FC (UGH: 44 mm vs. 53 mm, *p* = 0.002; LH: 60 vs. 65 mm, *p* = 0.021).

**Conclusions:**

This is the first report on the use of MRI to measure the performance of FC. Our study demonstrates that FC effectively repositioned the organs involved in POP.

## Introduction

Pelvic organ prolapse (POP) is one of the most common diseases in postmenopausal women and occurs in 41% and 38% of patients with and without a uterus, respectively.^1^ Pessaries, which are inserted into the vagina to support the uterus or vaginal wall, are the primary conservative treatment for POP^2^; however, a 5‐year prospective study demonstrated that 12% of pessary users experienced complications, such as pain or discomfort, vaginal erosion or bleeding, and constipation.^3^ Up to 28.5% and 4% of patients elected for surgery or no further treatment, respectively, after the cessation of pessary use.^3^

**FIGURE 1:**
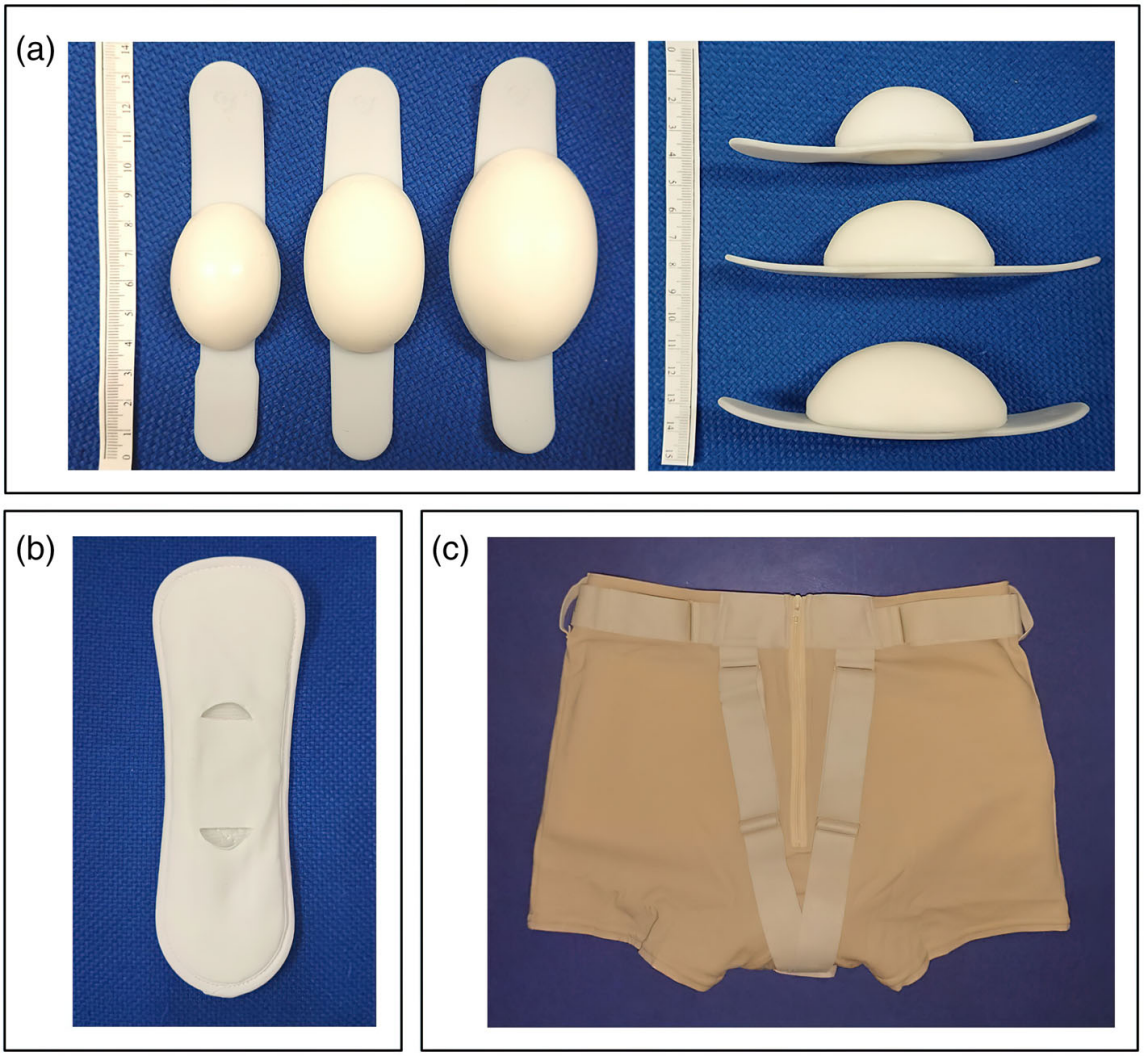
The three components of the FemiCushion device. (a) The cushions, which are available in three different sizes; (b) the cushion holder; and (c) the cushion supporter

FemiCushion (FC, Woman's Medical Research Inc., Tokyo, Japan) is a supportive device (supportive underwear) that treats all types of POP. It repositions prolapsed organs into the vaginal cavity with a hemispherical silicone cushion and is secured with special underwear (Figure 1). In recent years, FC has been widely and clinically recognized as a conservative treatment for POP. FC causes less damage to prolapsed organs and vaginal mucosa. It can be used by women who do not tolerate pessaries because of pain, erosion, or repeated pessary escape. It may also be used as an alternative to a pessary during the waiting period before surgery.

While some reports have demonstrated the clinical usefulness of FC,^4^, ^5^ the ability of FC to reposition prolapsed organs has not been objectively evaluated with imaging. Imaging is an important complementary tool in the assessment of pelvic floor disorders. Magnetic resonance imaging (MRI) can simultaneously evaluate all compartments of the pelvic floor and provide information about specific muscles and ligaments. Dynamic MRI as an auxiliary diagnostic imaging tool for POP has been reported.^6^, ^7^ Dynamic MRI of the pelvis during straining reproduces the prolapsed state under normal abdominal pressure and provides more accurate evaluations. Dynamic MRI can also be used to evaluate the effectiveness of repair by comparing preoperative and postoperative images.^8^, ^9^

This study utilized MRI findings to evaluate the anatomical changes induced by FC among patients with severe POP. We also assessed changes in the patient's prolapse quality of life (P‐QOL) scores to determine whether FC use alleviated patient symptoms. We hypothesized that FC effectively elevated the most prolapsed points of the involved organs and improved patients' P‐QOL scores.

## Methods

This prospective study included patients with POP quantification (POP‐Q) stage 3 or 4 who used FC for at least 1 month and underwent diagnostic MRI for the routine examination of POP at our institution between July 2020 and June 2021.

The FC supporters provided by us and used in this study had no metal components, which allowed the FC to be worn during MRI. Patients were allowed to wear their supportive underwear purchased at their own cost except when undergoing MRI. Patients were started on a small cushion size, and the size was increased to medium or large depending on the fit.

Patients underwent MRI with FC applied approximately 1 month after the FC fitting. The conditions for MRI with FC applied were similar to those for diagnostic MRI performed before FC application. The examiner confirmed the absence of symptoms, such as skin color changes and pain at the site, before and after the MRI. The FC was applied by the examiner prior to the MRI, and the patient confirmed that it was well‐positioned in both the standing and supine positions. Patients were asked to refrain from voiding their bladders 1 h before the MRI, and gel or contrast agent was not inserted into the vagina or rectum to avoid slippage of the FC. All patients underwent imaging in the supine position with a 1.5 T MR scanner (Optima MR450w; GE Healthcare, Chicago, IL, USA). An anatomic reference was acquired with 3D‐T2‐weighted static MRI sequences in the axial, sagittal, and coronal planes. The acquisition parameters of the 3D‐T2 scan were as follows: echo time (TE), 90 ms; repetition time (TR), 2000 ms; field of view (FOV), 25 cm; matrix, 256 × 256 mm^2^; slice thickness, 1.4 mm; total scan time, 9.5 min. Dynamic MR images were constructed from the midsagittal MRI using fast imaging employing steady state acquisition (FIESTA). The acquisition parameters of the FIESTA dynamic MRI were as follows: TE, 1.7 ms, minimum; TR, 5.2 ms; FOV, 35 cm; matrix, 256 × 256 mm^2^; interval, 2 s; slice thickness, 10 mm; total scan time, 2 min. Images were obtained between rest and maximal straining, and the patients were instructed by the radiological technologists. All MR images were analyzed by a single researcher.

**FIGURE 2:**
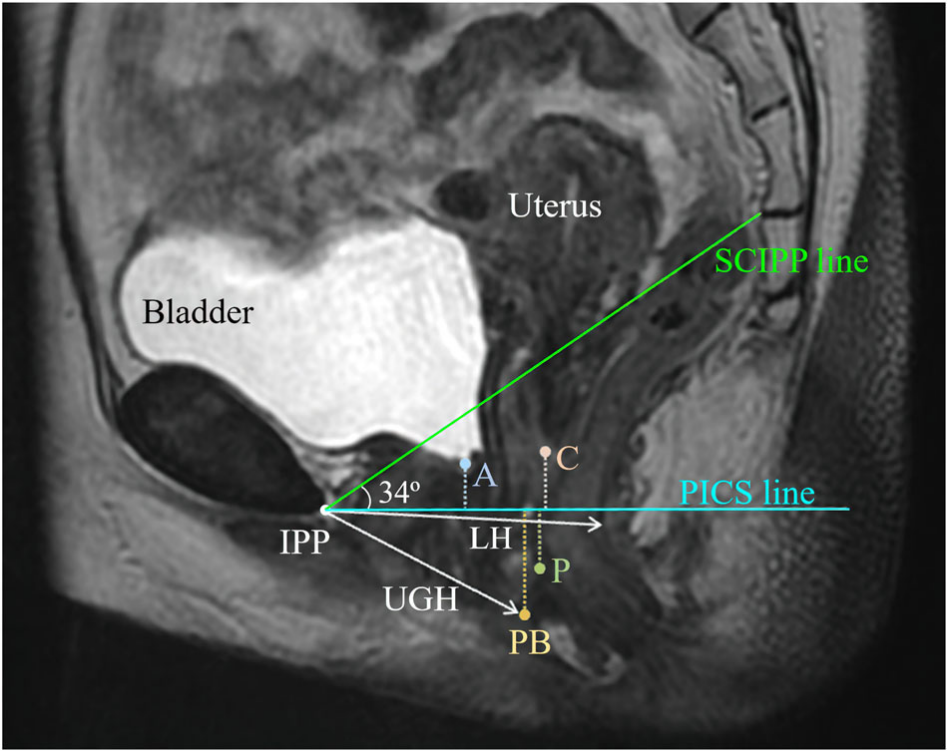
Measurements made in the midsagittal plane during magnetic resonance imaging (MRI). A, lowest point of the anterior vaginal wall; C, lowest point of the uterine cervix or vaginal stump, P, lowest point of the posterior vaginal wall, PB, front edge of the perineal body, SCIPP line, sacrococcygeal inferior pubic point line; PICS line, pelvic inclination correction line; LH, levator hiatus; UGH, urogenital hiatus

The lowest points of the anterior (A) and posterior (P) vaginal walls, uterine cervix or vaginal stump (C), and front edge of the perineal body (PB) were identified in the midsagittal plane (Figure 2). The Pelvic Inclination Correction System (PICS) line on the dynamic MRI midsagittal plane was used to evaluate the locations of the lowest points of these reference points.^10^ The PICS line was obtained by rotating the sacrococcygeal inferior pubic point line 34° clockwise. The PICS line (x‐axis) was oriented perpendicularly to the body axis (y‐axis), which provided the same coordinate system and allowed imaging data to be compared among patients.^10^ In the present study, the location of each point was defined as the vertical distance between the reference point and the PICS line. Positive and negative values denoted positions above (cephalad) and below (caudal) the PICS line, respectively (Figure 2).

We also measured the lengths of the urogenital (UGH) and levator hiatus (LH) in the dynamic MRI midsagittal plane, as described by Sammarco et al. (Figure 2).^11^ UGH was defined as the shortest distance from the pubic bone to the ventral aspect of the perineal body. LH was defined as the shortest distance from the inferior pubic point to the ventral surface of the levator ani. The LH corresponded to the line of action of the puborectal muscle.^12^

MRI was performed with and without the FC, and all measurements were compared between the two examinations. The difference between the measurements with and without the FC was calculated by subtracting the measurement without the FC from the one with the FC. The values were reported as ΔA, ΔC, ΔP, and ΔPB.

The POP‐Q system was used to quantify the vaginal compartments and stage POP before and approximately 1 month after FC use.^13^

The P‐QOL questionnaire was completed to assess the effect of POP symptoms on patient's quality of life before and approximately 1 month after FC use.^14^

Primary outcome measures were the changes in MRI measurements between images taken with and without FC. Secondary outcome measures included the changes in POP‐Q and P‐QOL before and after FC usage, as well as complications.

Statistical analysis was performed using JMP Pro version 15 (SAS Institute Inc., Cary, NC, USA). Wilcoxon signed‐rank tests were utilized to determine the differences between measurements with and without the FC in position. Spearman's correlation coefficients were utilized to compare the symptom score and the measured values on MRI. Statistical significance was set at a *p* value <0.05.

This study was approved by the Institutional Ethics Committee (registration number, 20‐H013) and complied with the requirements under the Declaration of Helsinki. Written informed consent was obtained for participation in the study.

## Results

Twelve patients were included in this study. The median age was 72 (range, 56–84) years, and the median body mass index was 24 (range, 18.7–31.8) kg/m^2^. Two patients had a previous total hysterectomy. Six cases of anterior vaginal wall prolapse were noted, as well as one case each of apical and anterior and posterior vaginal wall prolapse and two cases each of apical and anterior vaginal wall prolapse and complete eversion. Nine and three patients had stage 3 and 4 disease, respectively (Table 1).

**TABLE 1:** Baseline patient characteristics

Characteristics	*n* = 12
Age (years)^a^	72 (56–84)
Race^b^	
Japanese	12 (100)
Parity^a^	2 (1–3)
BMI (kg/m^2^)^a^	24.0 (18.7–31.8)
Menopausal^b^	12 (100)
Hormone replacement therapy usage^b^	0 (0)
Prior hysterectomy^b^	2 (17)
Prior POP surgery^b^	0 (0)
Prior continence surgery^b^	0 (0)
Hypertension^b^	6 (50)
Diabetes^b^	0 (0)
Chronic cough^b^	1 (8)
Smoking^b^	0 (0)
Baseline POP‐Q stage^b^	
III	9 (75)
IV	3 (25)
Predominant compartment of prolapse	
Complete eversion^a^	2 (22)
Apical^a^	1 (11)
Anterior^a^	6 (50)
Anterior and apical^a^	2 (22)
Apical and posterior^a^	1 (11)

Abbreviations: BMI, body mass index; POP‐Q, pelvic organ prolapse quantification.

^a^ Data expressed as median (range).

^b^ Data expressed as *n* (%).

One patient with complete eversion discontinued the FC while waiting for definitive surgery because of the discomfort caused by cushion contact with the prolapsed organ. The other 11 patients did not note any discomfort or adverse effects associated with the FC.

Four patients received definitive surgical treatment, which had been planned prior to their participation in this study, after using the FC for 1–5 months. Two patients underwent laparoscopic sacrocolpopexy, whereas one patient each received transvaginal mesh surgery and colpocleisis. One patient elected to discontinue the FC at 4 months and switched to a pessary. The remaining seven patients were satisfied with the FC because it allowed them to avoid surgery and pessary use. These seven patients continued to use the FC.

**FIGURE 3:**
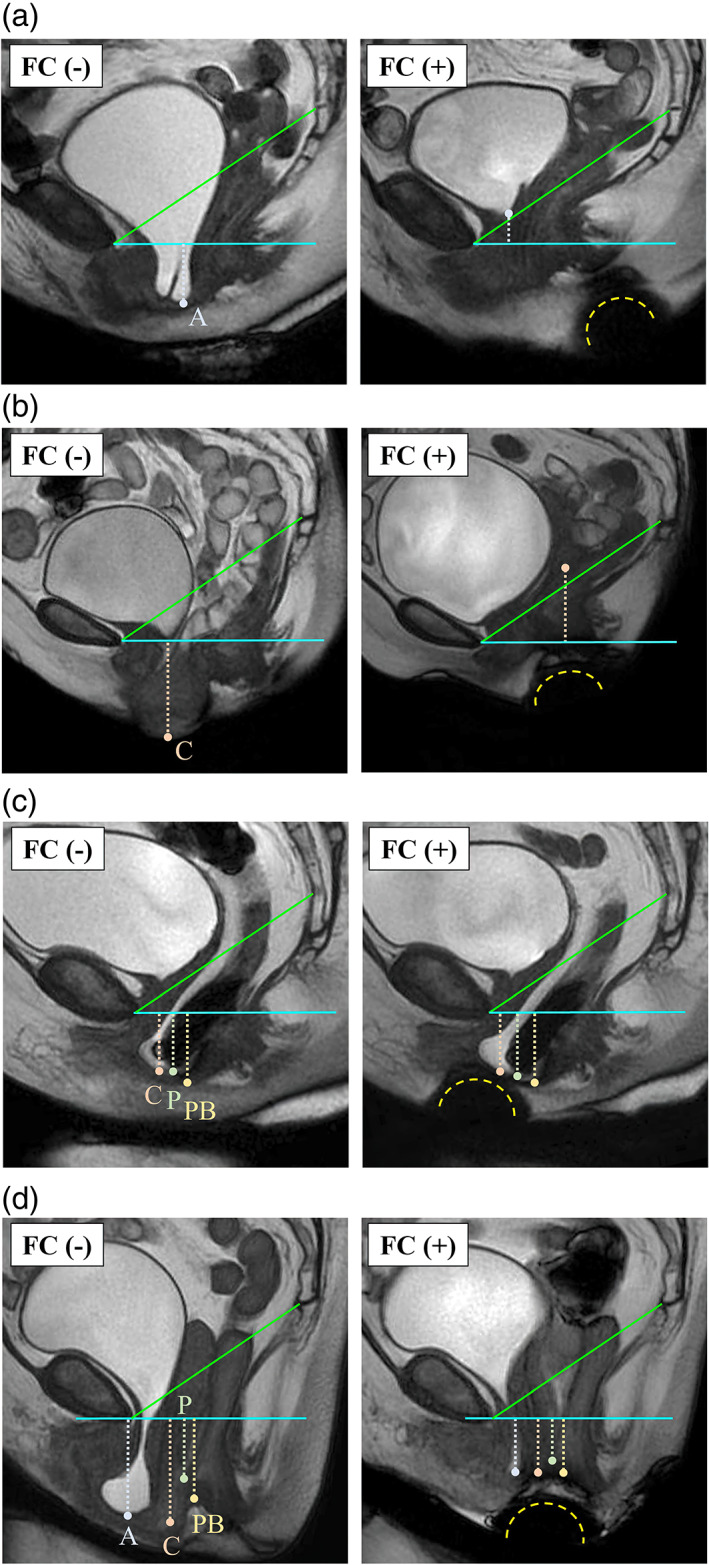
Examples of MRI measurements. The images depict (a) cystocele, (b) uterine prolapse, (c) enterocele and rectocele, and (d) complete eversion. The cushion was identified as a low‐intensity hemisphere below the perineum (yellow‐dashed lines)

**FIGURE 4:**
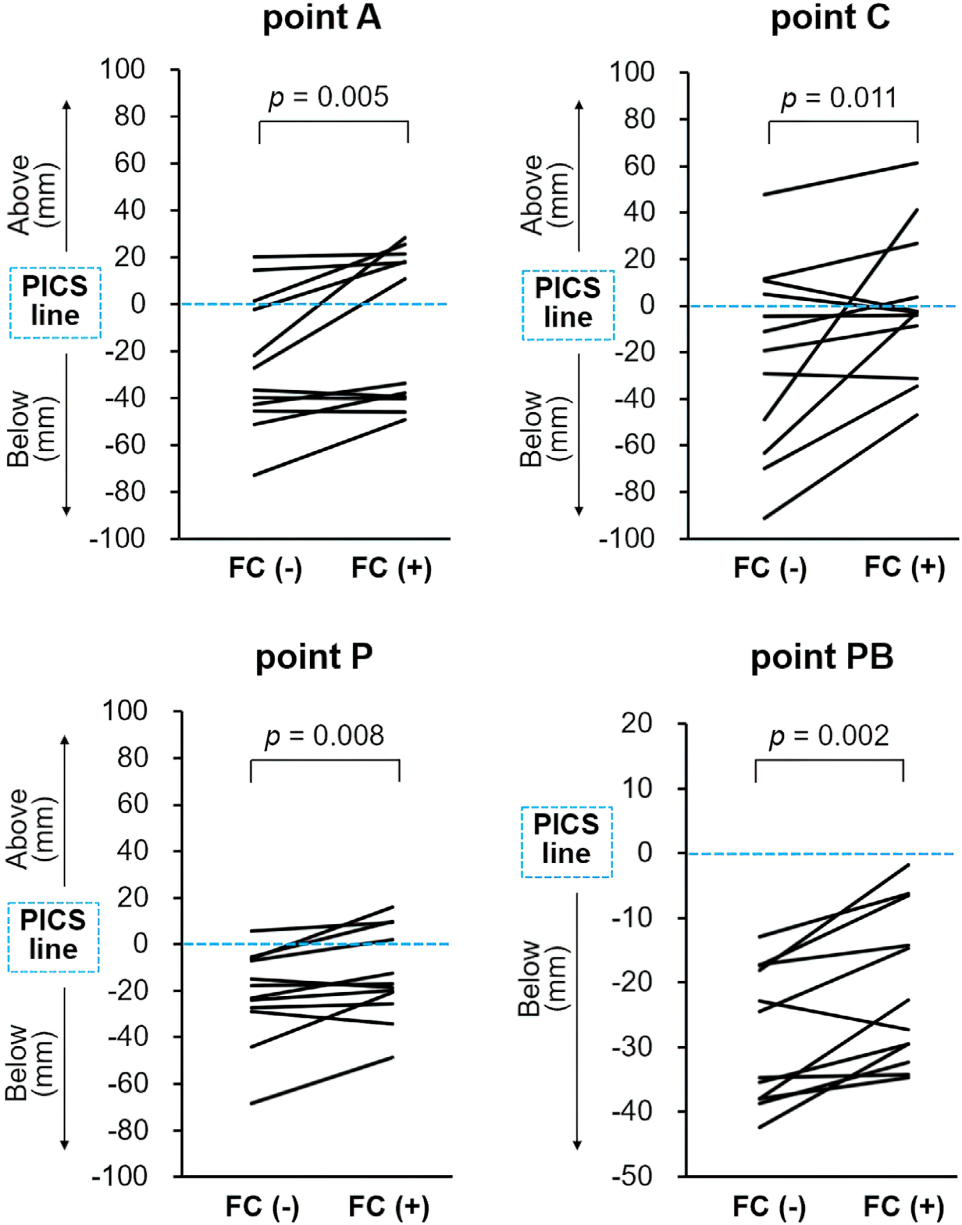
Organ displacements with and without the FemiCushion. The lines depict the changes in the positions of the lowest point of each organ relative to the PICS line with and without the FemiCushion in position. FC (−), without FemiCushion; FC (+), with FemiCushion

On T2‐weighted MRI, the FC was clearly identified as a low‐intensity hemisphere below the perineum in all patients (Figure 3). The lowest positions of all prolapsed organs were significantly higher with the FC in position than without the FC (median ΔA, 11 mm, *p* = 0.005; ΔC, 14 mm, *p* = 0.011; ΔP, 6 mm, *p* = 0.008; ΔPB, 7 mm, *p* = 0.002) (Figure 4).

**FIGURE 5:**
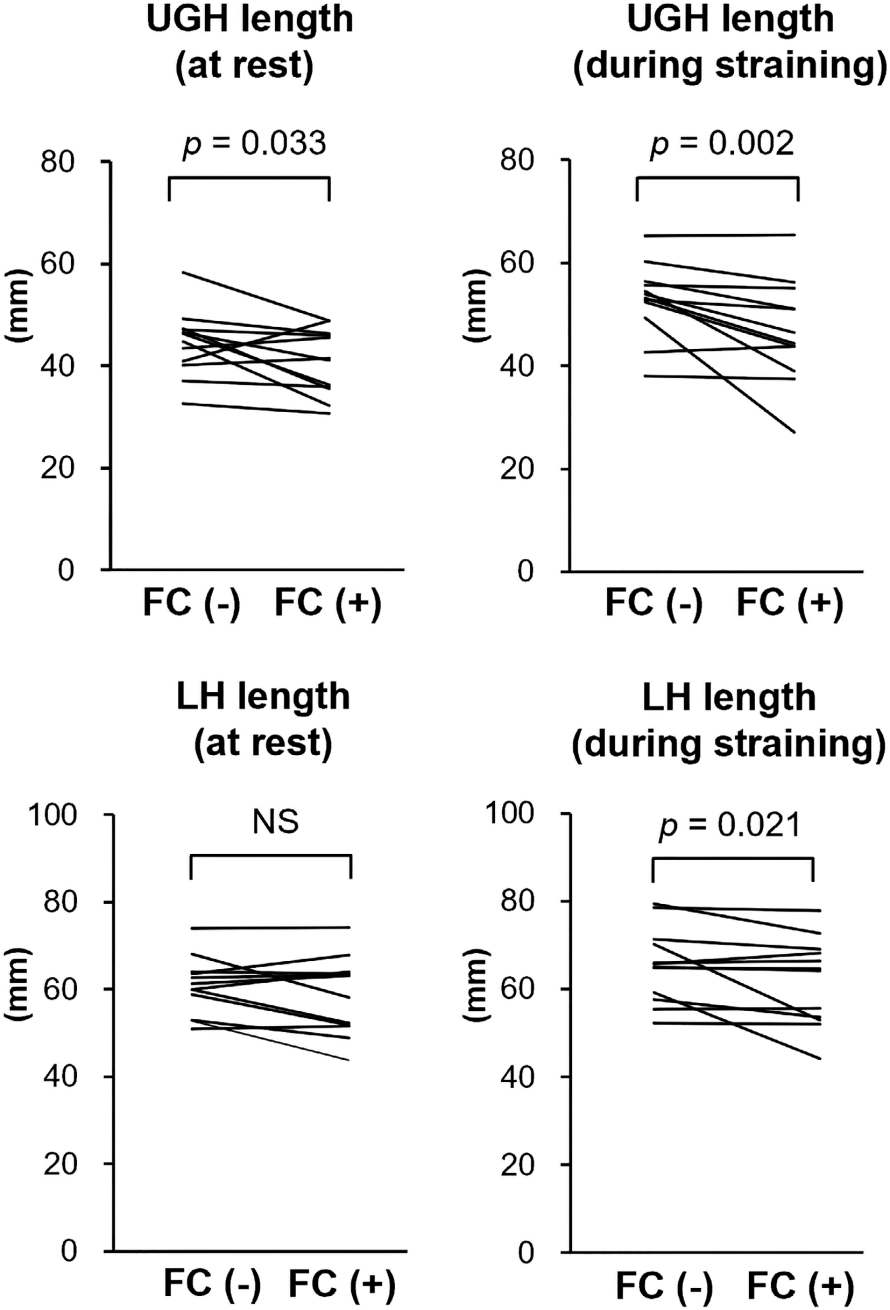
Comparison of the lengths of the levator hiatus (LH) and urogenital hiatus (UGH) with and without the FemiCushion in position. NS, not significant

The median UGH lengths at rest and upon straining with the FC were significantly shorter than those without the FC (at rest: 39 [range, 31–49] mm vs. 46 [range, 33–58] mm, *p* = 0.03; upon straining: 44 [range, 27–66] mm vs. 53 [range, 38–65] mm, *p* = 0.002) (Figure 5).

The median LH length upon straining was significantly shorter with the FC than without the FC (60 [range, 44–78] mm vs. 65 [range, 52–79] mm, *p* = 0.021). There was no significant difference in the mean LH length at rest with or without FC (Figure 5).

The POP‐Q stages before and 1 month after daily FC use were compared (Table 2). The measurements demonstrated improvements at the anterior vaginal wall (points Aa and Ba) and the apex (point C) after using the FC than before using the FC. No significant differences were observed in the other defined landmarks (Ap, Bp, D, Gh, Pb, and Tvl).

**TABLE 2:** The POP‐Q measurements before and 1 month after daily FC use

POP‐Q point	Number of patients	Before use	After use	*p*‐Value
		Median (range)	Median (range)	
Aa	12	3 (−1 to +3)	2 (−2 to +3)	0.008
Ba	12	4 (+2 to +6)	2 (−2 to +6)	0.039
C	12	2 (−3 to +9)	−1.5 (−4 to +6)	0.031
Gh	12	4 (+2 to +6)	4 (+2 to +5)	0.066
Pb	12	3.5 (+2.5 to +4)	3.5 (+3 to +4)	0.402
Tvl	12	8 (+7 to +9)	7 (+6 to +10)	0.156
Ap	12	−1 (−2 to +3)	−0.5 (−3 to +3)	0.617
Bp	12	0 (−2 to +3)	0 (−3 to +6)	0.617
D	10	−3.5 (−6 to +6)	−3.5 (−6 to +6)	0.188

**FIGURE 6:**
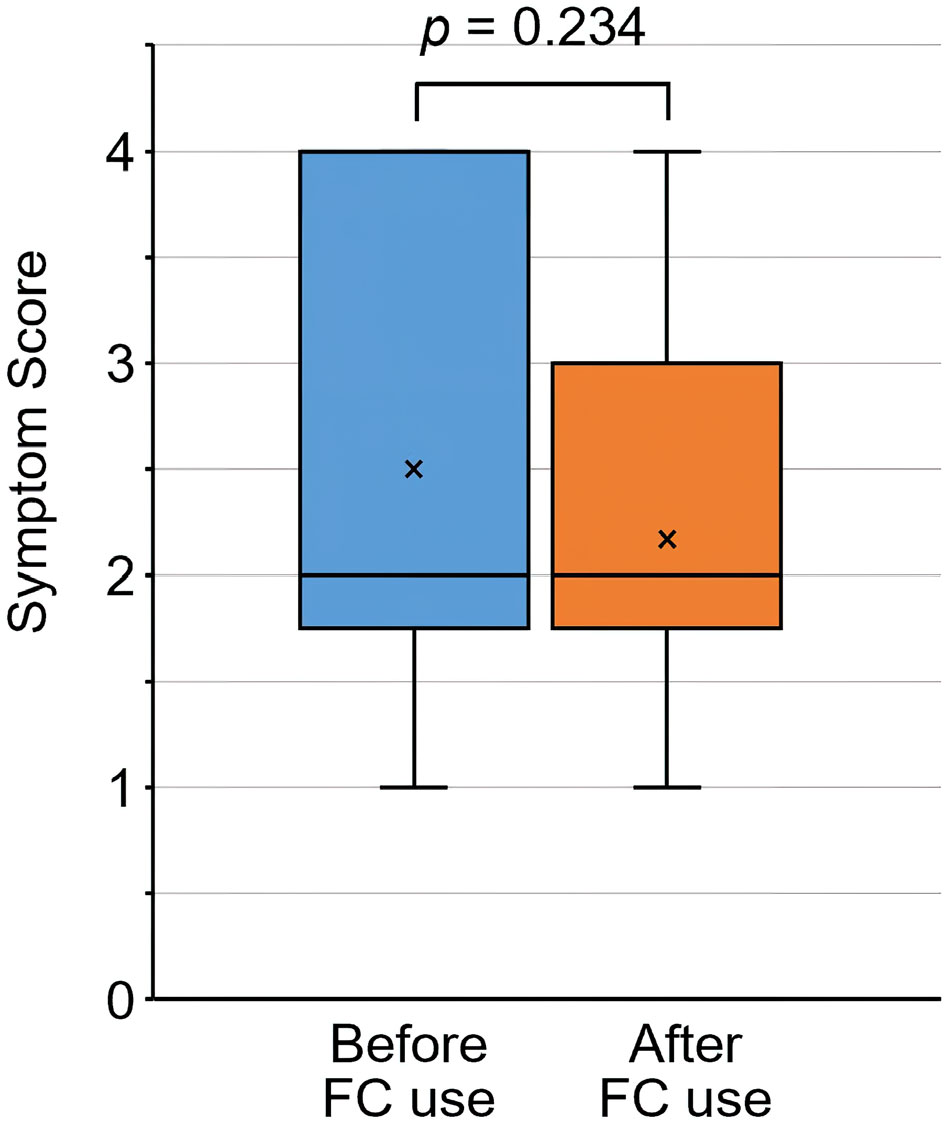
Comparison of the symptom score for the question “Pain or discomfort due to the POP,” in the prolapse quality of life (P‐QOL) questionnaire before and after FemiCushion use. The meaning of each score; 1 = Never, 2 = Sometimes, 3 = Often, 4 = All the time

There was no significant improvement in the overall evaluation of P‐QOL before and after the use of FC (data not shown). In the question “pain or discomfort due to POP,” the mean score decreased after FC use compared to before FC use (2.50 ± 1.19, 2.17 ± 0.90, respectively), although it was not significant (*p* = 0.234) (Figure 6). After using FC, the symptom scores were improved in five patients, remained unchanged in five patients, and worsened in two patients. No significant correlation was found between the degree of improvement in the score and ΔA, ΔC, ΔP, or ΔPB.

## Discussion

Pessaries are a conservative treatment option for patients with POP, but they may injure the vaginal walls. In contrast, FC is not inserted into the vagina, which allows patients to avoid the side effects often associated with pessary use, such as bleeding, vaginal wall erosion, and pain. Several reports have demonstrated the clinical usefulness of FC. Souto et al. reported improved vaginal symptoms and quality of life after 3 months of FC use in five women with POP.^4^ Kato et al. reported improvements in terms of the descending sensation associated with prolapse, voiding difficulty, and urinary frequency, which occurred in 80%, 40%, and 15% of the women, respectively.^5^ However, the ability of the FC to elevate prolapsed organs as reported has not been objectively evaluated. To our knowledge, this is the first study to utilize dynamic MRI to describe the anatomical changes induced by the FC.

In contrast, several reports have examined the anatomical changes associated with pessary use in patients with POP. Handa et al. conducted a retrospective study that demonstrated significant improvements in the POP‐Q stage of 18 patients who used a pessary for 1 year. The therapeutic effect of supportive pessaries has been attributed to preventing skeletal muscle strength loss from prolonged passive stretching.^15^ Jones et al. demonstrated a significant decrease in the genital hiatus size of 42 patients with POP after 3 months of pessary use. They further suggested that continued pessary use may result in some degree of recovery in the levator ani.^16^ Based on transperineal ultrasound images of the levator hiatal area, Manzini et al. reported that 3 months of pessary use was associated with changes in the puborectalis muscle (PRM) function of women with POP. They further proposed that vaginal pessaries might reduce the need for continuous contraction to support POP, which would allow the PRM to relax. It was unclear whether the observed changes could really be interpreted as a regain of PRM function.^17^ These reports suggest that continuous conservative treatment of POP may prevent progression of pelvic organ descent.

We hypothesized that FC usage can achieve the same results as a pessary without the risks associated with pessary use, such as vaginal erosion and bleeding. In this study, we noted significant elevation of three sites, namely the apex of the vagina and anterior and posterior vaginal walls, during straining with the FC, which suggested that the FC might be effective for any type of POP. However, while the FC provided an additional height of 2.5–3.5 cm, the median difference in elevation at each point was only approximately 1 cm. We theorized that the small difference in elevation was because of the severity of POP in our patients and because measurements were only taken in the midsagittal plane.

FC may be difficult to apply in some patients with severe POP. In these patients, the prolapsed organs should be digitally repositioned into the vagina prior to FC application; however, the FC may not completely sustain the repositioning in some cases. The involved organs may prolapse around the FC during straining.

All patients reported that the FC was well‐positioned and secure immediately before and after the MRI was performed with the patients in the supine position; however, our MRI data demonstrated that the FC was located outside the vagina and/or more inferiorly from the introitus than expected. We hypothesized that the effectiveness of the FC may be due to a combination of factors, such as the elevation of the prolapsed organs, as well as perineal support and reinforcement of the damaged pelvic floor. As such, we identified the locations of PB and measured the lengths of the UGH and LH. Elevation of the PB was considered to reflect perineal support, whereas longer UGH and LH lengths may indicate damage to the pubococcygeal and puborectal muscles, respectively.^11^ During straining, the PB of patients with FC was higher than that of patients without FC, which suggested that the PB was supported and elevated by the FC. The UGH length was significantly shortened at rest and during straining when the FC was positioned. This may indicate that FC provided forward support for the lower vagina. Furthermore, LH lengths were significantly shorter during straining with the FC than without the FC.

Nandikanti et al. measured UGH and LH lengths to compare the volumes of the levator ani bowl at rest and during straining in women with and without POP.^18^ They demonstrated that the average UGH and LH lengths during straining were 59 and 70 mm, respectively, in women with POP. These values were significantly longer than those of the controls (39 and 54 mm, respectively).

In this study, the median UGH and LH lengths during straining with the FC in position were 44 and 60 mm, respectively. These values were significantly shorter than those in the patients without the FC in position. FC use may shorten the UGH and LH to measurements similar to those observed in women without POP. Our results suggest that the FC not only directly elevates prolapsed organs but also supports the PB in an upward direction and closes the hiatuses.

In the POP‐Q, points Aa, Ba, and C were significantly improved after FC use, even though the FC was removed to take those measurements. This result was similar to that of a previous report, which showed improvement in POP‐Q score after pessary use.^15^ It was considered that the levator ani might be contracted due to the elevation and the return of the prolapsed organ into the vagina. Additionally, FC use for as little as a month might lead to improvement of POP. Significant shortening in LH and UGH during straining on MRI might support this conjecture about the therapeutic effect of FC use; however, it will be necessary to evaluate a larger number of cases with longer‐term FC use.

Improvements in the “pain or discomfort” symptom score in P‐QOL were noted in only 5 out of 12 patients. This result was considered to be due to the short period of use and was not related to the degree of change measured by MRI. The period of FC use in this study might be too short to obtain improvement in physical symptoms and quality of life. However, the primary outcome measures of this study were the MRI assessments with and without FC application, not the subjective improvements that would require additional research on changes after long‐term use.

In this study, only one patient with complete eversion discontinued FC use due to discomfort caused by cushion contact with the prolapsed organ. The other 11 patients did not report any discomfort or adverse effects associated with FC. Furthermore, Souto et al.^4^ reported no complications after 3 months of FC use, although the number of cases in that study was small. In another study of 20 patients, Kato et al.^5^ reported that 35% of the patients discontinued FC use after 2 months of use due to discomfort, bleeding, and/or difficulty to wear. However, this study might have had a higher incidence of FC‐related complications as it included older patients, as well as those who originally had complications. In addition, Sarma et al.^19^ reported adverse effects of long‐term pessary use. Although 167 of 273 patients were successfully using pessaries at 4 weeks, subsequently, 93 (56%) experienced complications, such as bleeding, extrusion, severe vaginal discharge, pain, and constipation. Therefore, we speculate that FC has fewer complications than pessaries, although there are no reports on long‐term FC use.

The study has several limitations, which include the small number of cases and short follow‐up period. It was not possible to evaluate the efficacy of the FC for each type of POP because of the small number of cases. The participants in this study had severe stage 3 or 4 POP. Four patients used FC while waiting for definitive surgical treatment. Our data should be verified in patients with all stages of POP to accurately evaluate the efficacy of the FC.

Moreover, the MR images analyzed in this study were taken in the supine position. This may have underestimated the position of the prolapsed organs during standing. Previous studies have demonstrated more significant prolapse during straining in patients with POP‐Q stage ≥2 in upright MRI compared to that in supine MRI.^20^ This study performed MRI in the supine position because no machines for upright MRI were available. Dynamic MRI clearly demonstrates organ prolapse during straining even in the supine position^21^; however, future studies should consider utilizing an upright MRI to reflect the effect of the FC in daily life.

In this study, some patients with severe POP demonstrated a significant reduction in the amount of prolapse following FC application, even if the anatomic changes measured in the midsagittal plane were small. A three‐dimensional coordinate system, which utilizes four bony landmarks, including the ischial spine, has been proposed to quantitatively assess the lateral anatomical supports of the pelvis^22^; however, it is difficult to obtain dynamic MR images of the midsagittal plane and ischial spine simultaneously.

To our knowledge, this is the first report to examine the effect of FC with MRI. Our study clearly demonstrated that all points of the involved organs were repositioned with FC use. UGH and LH lengths were also shortened during straining with the FC. These findings may explain why FC is effective. Further investigation with a larger number of cases and longer follow‐up periods is necessary to determine whether the FC provides anatomic support and improves the quality of life in patients with POP.

## Author Contributions

Yukiko Nomura contributed to the design of the study, the analysis and interpretation of the data, statistical analysis and writing of the manuscript. Yoshiyuki Okada, Chie Nakagawa and Ippei Kurokawa acquired the data and contributed to the statistical analysis. Hidefumi Fujisawa and Miwa Shigeta contributed to the interpretation of data and writing of the manuscript. Yasukuni Yoshimura contributed to the design of the study and supervised the process of writing the manuscript. All authors have read and approved the manuscript.

## Conflict of Interest

The authors declare no conflict of interest for this article.

## Data Availability

The data that support the findings of this study are available on request from the corresponding author. The data are not publicly available due to privacy or ethical restrictions.

## References

[1] Hendrix SL , Clark A , Nygaard I , Aragaki A , Barnabei V , McTiernan A . Pelvic organ prolapse in the Women's Health Initiative: gravity and gravidity. Am J Obstet Gynecol. 2002;186:1160–6. 12066091 10.1067/mob.2002.123819

[2] Bradley C . Pessaries and devices: non‐surgical treatment of pelvic organ prolapse and stress urinary incontinence. In: Cardozo L , Staskin D , editors. Textbook of female urology and urogynecology. 3rd ed. Colchester, UK: Informa Healthcare; 2010. p. 47.

[3] Lone F , Thakar R , Sultan AH , Karamalis G . A 5‐year prospective study of vaginal pessary use for pelvic organ prolapse. Int J Gynaecol Obstet. 2011;114:56–9. 21575953 10.1016/j.ijgo.2011.02.006

[4] Souto S , Palma T , Palma P . Femicushion™: a new pessary generation‐pilot study for safety and efficacy. Pelviperineology. 2016;35:44–6.

[5] Kato K , Suzuki S , Yamamoto S , et al. The effectiveness of supportive underwear for women with pelvic organ prolapse. Rinsho Hinyokika. 2010;64:761–5.

[6] Kobi M , Flusberg M , Paroder V , Chernyak V . Practical guide to dynamic pelvic floor MRI. J Magn Reson Imaging. 2018;47:1155–70. 29575371 10.1002/jmri.25998

[7] Pannu HK , Kaufman HS , Cundiff GW , Genadry R , Bluemke DA , Fishman EK . Dynamic MR imaging of pelvic organ prolapse: spectrum of abnormalities. Radiographics. 2000;20:1567–82. 11112811 10.1148/radiographics.20.6.g00nv311567

[8] Brocker KA , Alt CD , Rzepka J , Sohn C , Hallscheidt P . One‐year dynamic MRI follow‐up after vaginal mesh repair: evaluation of clinical, radiological, and quality‐of‐life results. Acta Radiol. 2015;56:1002–8. 25136056 10.1177/0284185114544241

[9] van IJsselmuiden MN , Lecomte‐Grosbras P , Witz J‐F , Brieu M , Cosson M , van Eijndhoven HW . Dynamic magnetic resonance imaging to quantify pelvic organ mobility after treatment for uterine descent: differences between surgical procedures. Int Urogynecol J. 2020;31:2119–27. 32277268 10.1007/s00192-020-04278-5

[10] Betschart C , Chen L , Ashton‐Miller J , DeLancey J . On pelvic reference lines and the MR evaluation of genital prolapse: a proposal for standardization using the pelvic inclination correction system. Int Urogynecol J. 2013;24:1421–8. 23640002 10.1007/s00192-013-2100-4 PMC3986860

[11] Sammarco AG , Nandikanti L , Kobernik EK , et al. Interactions among pelvic organ protrusion, levator ani descent, and hiatal enlargement in women with and without prolapse. Am J Obstet Gynecol. 2017;217(614):e1–614.e7. 10.1016/j.ajog.2017.07.007 PMC5671348 28709583

[12] Betschart C , Kim J , Miller JM , Ashton‐Miller JA , DeLancey JOL . Comparison of muscle fiber directions between different levator ani muscle subdivisions: in vivo MRI measurements in women. Int Urogynecol J. 2014;25:1263–8. 24832855 10.1007/s00192-014-2395-9 PMC4140951

[13] Bump RC , Mattiasson A , Bø K , et al. The standardization of terminology of female pelvic organ prolapse and pelvic floor dysfunction. Am J Obstet Gynecol. 1996;175:10–7. 8694033 10.1016/s0002-9378(96)70243-0

[14] Digesu GA , Khullar V , Cardozo L , Robinson D , Salvatore S . P‐QOL: a validated questionnaire to assess the symptoms and quality of life of women with urogenital prolapse. Int Urogynecol J. 2005;16:176–81. 10.1007/s00192-004-1225-x 15875234

[15] Handa V , Jones M . Do pessaries prevent the progression of pelvic organ prolapse? Int Urogynecol J Pelvic Floor Dysfunct. 2002;13:349–52. 12466904 10.1007/s001920200078

[16] Jones K , Yang L , Lowder JL , et al. Effect of pessary use on genital hiatus measurements in women with pelvic organ prolapse. Obstet Gynecol. 2008;112:630–6. 18757662 10.1097/AOG.0b013e318181879f

[17] Manzini C , van den Noort F , Grob ATM , Withagen MIJ , van der Vaart CH . The effect of pessary treatment on puborectalis muscle function. Int Urogynecol J. 2021;32:1409–17. 33847771 10.1007/s00192-021-04766-2 PMC8042456

[18] Nandikanti L , Sammarco AG , Chen L , Ashton‐Miller JA , DeLancey JO . Levator bowl volume during straining and its relationship to other levator measures. Int Urogynecol J. 2019;30:1457–63. 31222569 10.1007/s00192-019-04006-8 PMC7836809

[19] Sarma S , Ying T , Moore KH . Long‐term vaginal ring pessary use: discontinuation rates and adverse events. BJOG. 2009;116:1715–21. 19906018 10.1111/j.1471-0528.2009.02380.x

[20] Grob ATM , Heuvel JO , Futterer JJ , et al. Underestimation of pelvic organ prolapse in the supine straining position, based on magnetic resonance imaging findings. Int Urogynecol J. 2019;30:1939–44. 30656361 10.1007/s00192-018-03862-0 PMC6834735

[21] Comiter CV , Vasavada SP , Barbaric ZL , Gousse AE , Raz S . Grading pelvic prolapse and pelvic floor relaxation using dynamic magnetic resonance imaging. Urology. 1999;54:454–7. 10475353 10.1016/s0090-4295(99)00165-x

[22] Reiner CS , Williamson T , Winklehner T , et al. The 3D pelvic inclination correction system (PICS): a universally applicable coordinate system for isovolumetric imaging measurements, tested in women with pelvic organ prolapse (POP). Comput Med Imaging Graph. 2017;59:28–37. 28609701 10.1016/j.compmedimag.2017.05.005 PMC5526449

